# Causes of discomfort in the academic workplace and their associations with the different burnout types: a mixed-methodology study

**DOI:** 10.1186/1471-2458-13-1240

**Published:** 2013-12-30

**Authors:** Jesús Montero-Marín, Javier Prado-Abril, José Miguel Carrasco, Ángela Asensio-Martínez, Santiago Gascón, Javier García-Campayo

**Affiliations:** 1 Department of Psychiatry, University of Zaragoza, Zaragoza, Spain; 2 School of Health and Sports, University of Zaragoza, Huesca, Spain; 3 Department of Psychology, University of Zaragoza, Teruel, Spain; 4 Psychiatry Service, Miguel Servet University Hospital, Zaragoza, Spain; 5 Research Unit, Spanish Rheumatology Society, Madrid, Spain; 6 REDIAPP “Research Network on Preventative Activities and Health Promotion” (RD06/0018/0017), Zaragoza, Spain

**Keywords:** Burnout subtypes, University, Workplace discomfort, BCSQ-36, Qualitative and quantitative analysis, Mixed-methodology

## Abstract

**Background:**

Burnout is the result of prolonged workplace exposure to chronic stress factors and may present itself in one of the following subtypes: “frenetic”, “under-challenged” and “worn-out”. The aims of the present study were to identify the causes of workplace discomfort that affect employees in large organizations and to determine the predictive power of these causes with regard to the burnout subtypes.

**Method:**

We employed a qualitative and quantitative analysis (QQA), using a cross-sectional design with an online survey administered to a randomly selected sample of University workers (n = 409). To determine the causes of discomfort, we raised the following open question: *“What aspects of your work generate discomfort for you?*”. The responses were subjected to content analysis and categorized by three independent referees. The concordance between the responses was estimated with the kappa coefficient (k). Subtype classification was assessed according to the “Burnout Clinical Subtype Questionnaire” (BCSQ-36). The degree of association between the motives for the complaint and the burnout profiles was evaluated using adjusted odds ratio (OR), which was based on multivariate logistic regression models.

**Results:**

The causes of discomfort included: physical environment (setting aspects, material conditions, journey/access), organization (schedules, structure, functions, interpersonal relations) and individual conditions (workload, powerlessness, rewards, negligence). The concordance index between the referees was k = 0.80. Employees who were upset with the hierarchical structure were more likely to be classified as frenetic (OR = 4.32; 95% CI = 1.43-13.06; p = 0.010); those who complained of routine duties were more likely to be classified as under-challenged (OR = 5.33; 95% CI = 1.84-15.40; p = 0.002); those whose discomfort was caused by structure control systems were more likely to be classified as worn-out (OR = 6.13; 95% CI = 1.57-23.91; p = 0.009).

**Conclusions:**

The causes of discomfort among the different burnout subtypes are primarily attributable to the organization itself, in response to the structure and functions. The associations observed between the different subtypes and motives for complaint are consistent with the clinical profile-based syndrome definition, which suggests that interventions should be case-specific.

## Background

Since the first study on burnout was conducted a few decades ago [1], the interest in this syndrome has grown significantly. This is understandable in the context of the structural-level transformations experienced by Western societies, which resulted from the thrust exerted by economic forces. The impact of all these changes has significantly influenced employee workplace conditions and has created higher stress vulnerability [2,3].

Burnout is a consequence of prolonged exposure to chronic workplace stress and is typically defined by the following dimensions: exhaustion, cynicism and inefficacy [2,4,5]. Exhaustion is a feeling of not being able to give more of oneself at the workplace, cynicism refers to a lack of interest and loss of meaning of the job and inefficacy is the feeling of a lack of competence resulting from not performing workplace tasks correctly. This phenomenon is likely to occur in any work context and is characterized by a general state of fatigue, the use of a depersonalized coping style and a stark decrease in professional performance, tendencies which reflect huge discrepancies between the person and their work [5,6]. This syndrome definition has been widely accepted [7-10], although there is no unanimous agreement on the number of constituent dimensions [11,12]. For some authors, exhaustion is the central aspect of burnout and its clearest manifestation, as it reflects the links between stress and the syndrome [13-15]. However, it does not clarify the key aspects of the interaction between the person and their work environment and thus should perhaps be considered as a necessary but insufficient criterion [6].

This definition has some psychometric weaknesses, which require that it be used with caution [16]. We should add that the definition was not developed on the basis of clinical observation nor was it founded on a systematic theorization of the syndrome; instead, it was proposed inductively by a factorial grouping of a set of rather arbitrary items [17]. This definition also does not clarify the relationships between the constituent components and raises no possible antecedents or consequences of the disorder; thus, it lacks a theoretical structure [18-20]. Therefore, some authors have embarked upon the task of discovering the antecedent factors of burnout [21], with the assumption that a complete understanding of the syndrome etiology could facilitate early recognition [22,23].

The understanding of burnout as a unitary phenomenon with relatively consistent symptoms and etiology in all individuals has also been heavily criticized [24]. Alternatively, three different burnout profiles have been proposed according to a phenomenological approach based on clinical observation. These profiles are explained in terms of the following subtypes: ‘frenetic’, ‘under-challenged’ and ‘worn-out’, each of which presents a unique way of coping with workplace discomfort [25,26]. The frenetic subtype refers to the category of individuals who work ever harder until exhaustion while seeking success commensurate to the level of tension caused by their efforts. The under-challenged subtype refers to the workers who experience non-stimulating work conditions that do not provide the necessary satisfaction. The worn-out subtype consists of people who easily give up when faced with stress or the absence of rewards.

The frenetic subtype is characterized by great ‘involvement’, ‘ambition’ and ‘overload’. Involvement means investing all necessary effort to overcome difficulties; ambition is the need to secure great triumphs and achievements; and overload is the risking of health and abandoning of personal life in favor of pursuing good results. The features of the under-challenged subtype are ‘indifference’, ‘boredom’ and ‘lack of development’. Indifference is defined as not caring and showing little interest in or enthusiasm for the tasks; boredom is defined as experiencing work as something mechanical, routine and with little variety in activities; and the lack of development is defined as the desire to engage in other work where the worker can better develop his or her abilities. The worn-out subtype is characterized by ‘neglect, ‘lack of control’ and ‘lack of acknowledgement’. Neglect describes negligence in response to difficulties; lack of control describes the feeling of being powerless as a result of dealing with situations that are beyond one’s control; and lack of acknowledgement describes the belief that effort and dedication are not taken into account by the organization in which the employee works [27].

Social exchange theories consider burnout to be both an individual and an environmental disorder because workplaces determine the ways in which employees perform their tasks [28]. Based on this approach, the origins of burnout can be found in the perceived lack of reciprocity experienced by some individuals in social comparison and exchange processes due to the feeling that they will lose or will not be able to recover their investments [29,30]. People are motivated to obtain and protect their resources, which is why when imbalances are perceived between investments made and benefits obtained in return, people will react by trying to re-establish the lost equilibrium. Each of the burnout subtypes uniquely perceives inconsistencies between work investment and the obtained rewards [31], and thus, the level of dedication to the tasks is adjusted in an attempt to balance the perceived inequity [27]. In this context, the dedication of the frenetic type is high, given the great involvement with which this burnout profile tries to maximize rewards. The involvement of the under-challenged subtype becomes intermediate, owing to the indifference towards work that does not provide personal satisfaction. Meanwhile, the involvement of the worn-out subtype is low as a result of abandonment, with which this subtype tries to minimize efforts. Therefore, as a typological classification criterion, the degree of dedication towards tasks conceptually integrates the differential proposal of burnout, as influenced by social exchange theories.

The definition of burnout, which is based on clinical profiles, has demonstrated adequate psychometric properties in workers and students [32-35]. Furthermore, socio-demographic and general occupational aspects have been observed to permit the establishment of differences between the subtypes. For example, the proportion of the frenetic subtype increases in direct relation to weekly work hours; the proportion of the under-challenged subtype is greater in bureaucratic occupations and among administrative staff; and the proportion of the worn-out subtype increases with the length of time worked at the same organization [3]. This interpretation of the syndrome raises the possibility of developing new interventions that are adjusted to the specific dysfunctions of each profile, with the understanding that each could be affected by different causes of discomfort. The frenetic burnout subtype could suffer from barriers that prevent him or her from expressing ambition; the under-challenged subtype could be affected by all aspects that impede his or her personal development at the workplace; and the worn-out subtype could suffer due to his or her negligent attitude.

We have already mentioned that burnout is a consequence of interactions between the individual and the environment. However, we have not yet determined in what way these interactions operate differentially in the distinct burnout profiles. This might be due to the methodological complexity of including all possible types of discomfort that may present themselves at the individual level in large work organizations. Therefore, the objectives of this study were as follows: to investigate the causes of workplace discomfort among the employees of large companies in their own words and to examine the association between these causes of discomfort and the various subtypes of burnout.

## Method

### Design

We used a cross-sectional design with a self-administered online survey. To achieve a broad understanding of all participants and the complexities of their environments resulting from multiple and different individual situations, a mixed or qualitative and quantitative (QQA) analysis methodology was used. QQA offers a research approach that combines qualitative and quantitative techniques, thus overcoming the limitations of both approaches and introducing a holistic model capable of integrating complex contexts [36].

### Participants

We recruited a multi-occupational group of workers in diverse positions; all employees of the University of Zaragoza who were employed as of January 2008 (N = 5,493) were used as the universal reference population. This population is at a high risk of burnout development, because it is composed of professionals who work “face to face” with other people [16]. The necessary sample size was calculated to achieve estimates with a confidence interval of 95% and a margin for error of 3.5%, while assuming a burnout prevalence of 18% [37]. This calculation resulted in a sample size of 427 individuals. The approximate response rate obtained through web-mail surveys is usually 27% [38,39]. Therefore, we selected 1,600 individuals by stratified random sampling, which was dependent on occupation (58% of teaching and research staff or TRS, 33% of administrative and service staff or ASS, and 9% interns or INT) and based on an alphabetically ordered list of the entire workforce. The sample size calculation and randomization of individuals were performed with Epidat 3.1 software.

### Procedure and ethics

In February 2008, a detailed e-mail message was sent to the selected subjects that explained the objectives of the study, to whom it was directed, the voluntary nature of participation, the potential benefits and risks, and data confidentiality. This message contained a link to the online survey, with two keys that permitted access after providing informed consent. The participants received a report with an explanation of the results in return for their participation. The study was approved by the “Aragon Regional Ethical Committee”.

### Measures

#### Causes of workplace discomfort

To determine the causes of discomfort that affected the participants in their work environments, we asked the following open question: *“What aspects of your work generate discomfort for you?*” The participants could freely answer the question, in their own words, in an assigned space without word and time limits.

#### Socio-demographic and occupational characteristics

We asked about age, sex, stable relationships (‘yes’ vs. ‘no’), children (‘yes’ vs. ‘no’), educational level (‘secondary school or lower’, ‘university’, ‘doctorate’), occupation (TRS, ASS, INT), number of hours worked per week (‘<35′,’35-40′, ‘>40′), time served (‘<4 years’, ‘4-16 years’, ‘>16 years’), salary (‘<€1,200′, ‘€1,200-2,000′, ‘>€2,000′), time off in the previous year (‘yes’ vs. ‘no’), contract duration (‘permanent’ vs. ‘temporary’) and contract type (‘full-time’ vs. ‘part-time’) in a series of specifically prepared questions.

#### Burnout subtypes

To assess the burnout subtypes, we administered the “Burnout Clinical Subtype Questionnaire” (BCSQ-36) [32]. This questionnaire includes 36 items, which are distributed in 3 scales and 9 sub-scales of 4 items each. The frenetic subtype scale assesses the following dimensions: involvement (e.g., “I react to difficulties in my work with greater participation”), ambition (e.g., “I have a strong need for important achievements in my work”) and overload (e.g., “I overlook my own needs in order to fulfill work demands”). The under-challenged subtype scale evaluates indifference (e.g., “I feel indifferent about my work and have little desire to succeed”), lack of development (e.g., “My work doesn’t offer me opportunities to develop my abilities”) and boredom (e.g., “I feel bored at work”). The worn-out subtype scale probes neglect (e.g., “When things at work don’t turn out as well as they should, I stop trying”), lack of acknowledgement (e.g., “I think my dedication to my work is not acknowledged”) and lack of control (e.g., “I feel the results of my work are beyond my control”). Subjects had to indicate the degree to which they agreed with the presented sentences using a Likert-type scale, with 7 response options that were scored from 1 (“completely disagree”) to 7 (“completely agree”). The BCSQ-36 presents adequate psychometric features with values α ≥ 0.80 in all dimensions [32,35].

### Data analysis

We performed a descriptive analysis of the socio-demographic and occupational characteristics using means, standard deviations and percentages according to the natures of the variables.

#### Subject content

The participants’ answers to the open question were extracted from an independent database to facilitate analysis. One of the researchers (JMM) performed a subject content analysis (CA) to identify the emerging categories on which all of the answers could be coded [40]. First, the general frameworks of workplace discomfort were identified. Next, we attempted to determine which aspects or work conditions were typical of these frameworks. Finally, we detailed the conceptually integrated subject content into lower hierarchical levels. Along with another researcher (JPA), we empirically defined each of the emerging categories by discussing their abilities to adequately capture all of the answers. The appropriate adjustments were made by consensus to ensure that each definition would be comprehensive and exclusive of the others [41]. Three researchers (SG, JMC, AAM) independently reviewed the participants’ answers and assigned codes that corresponded to the segments that made up each answer (the answers could be coded with various categories according to the conceptual extent of the expressed subject content). The qualitative analysis of the data was conducted with Maxqda 2007 software.

#### Concordance

We determined the adequacy of the category system and the coding precision by calculating the generalized kappa [42] for each of the subjects emerging from CA. This statistic is an extension of the classic kappa coefficient, which allows it to be used for the case of three different coders and two observational possibilities (presence or absence of the feature), while still subtracting the possible concordance at random. We calculated the standard errors associated with this coefficient using the second-order Fleiss algorithm [43] and estimating confidence intervals at 95% (95% CI). We contrasted the kappa value to the behavior of the random classifier [44]. In general, a kappa value >0.40 is considered acceptable and >0.75 is believed to be excellent [45,46]. We compared the coefficients from the various codes with each other using a kappa homogeneity test, calculating the value of the global kappa and its 95% CI. We estimated the prevalence of the codes by calculating the percentages of positive assignments compared to the total assignments issued by all of the evaluators. We also calculated the percentage of agreement among the three referees. We coded the categories in which the three independent referees fully coincided, subsequently moving to the quantification of the text [41].

#### Estimating associations

We considered participants with scores above the 75th percentile (P_75_) in the scales of the BCSQ-36, as ‘high scores’, while those with scores below this percentile as ‘low scores’ [3,34,47,48]. We estimated the potential associations between the burnout subtypes and the causes of workplace discomfort from the CA by calculating the crude odds ratios (OR) with a CI of 95% based on a simple binary logistical regression (LR) analysis. The causes of discomfort were categorized as dichotomous variables according to their presence (1) or absence (0). The significance of these associations was assessed with the Wald test. Factors that demonstrated significant values in the bivariate analysis (p < 0.05) were included in the multivariate LR, which included sex and age variables, to estimate adjusted ORs with a CI of 95%. The significance of the adjusted ORs was evaluated with the Wald test. The adjustment of each multivariate model was assessed with a Hosmer-Lemeshow test, while the discriminative power was verified by the success rate and the area under the ROC curve of the function of the predicted probabilities and the status variable (high scores/low scores), with a cut-off of p = 0.5. All of these comparisons were bilateral with a significance level of α < 0.05. All quantitative analyses were performed with the SPSS-15 and Epidat 3.1 statistical software packages.

## Results

### Description of the participating sample

Overall, 409 participants responded, yielding a response rate (RR) of 25.6%. The mean participant age was 40.51 years (SD = 9.09), and 44.4% were males. The majority of participants (78.1%) were in a stable relationship, and 49.9% had children. Furthermore, 15.5% of the participants had achieved secondary or lower schooling, 52.1% held university degrees and 32.4% held doctorates. In terms of job position, 42.9% were ‘TRS’, 46.9% were ‘ASS’ and 10.2% were ‘INT’. With regard to work hours, 40.6% worked ‘<35 h per week’, 26.8% worked ‘35-40 h’ and 32.6% worked ‘>40 h’. In terms of employment duration, 18.5% had worked at the university for ‘less than 4 years’, 44.6% for ‘between 4–16 years’ and 36.9% for ‘more than 16 years’. The participant income distribution was as follows: 31.1% had a monthly income of ‘less than €1,200′, 42.1% of ‘€1,200-2,000′ and 26.8% of ‘more than €2,000′. Additionally, 67% of the participants had not taken sick leave in the previous year; 63.6% were permanent employees and the majority (93.8%) worked full time. Finally, 21.3% of the participants did not answer the open question.

### Sources of discomfort at the workplace

We uncovered three general frameworks of workplace discomfort: physical environmental conditions, organizational characteristics and individual conditions. Table 1 presents the hierarchical levels and the lower-level categories that resulted from the content analysis.

**Table 1:** Causes of discomfort in the work environment and concordance indices

	**%**	**k**	**SE**	**95% CI**	**prop**	**z**	**(p)**
**Physical environment**							
***Settings***							
Lighting	99.4	0.93	0.03	0.87 - 0.99	3.1	28.85	< 0.001
Air conditioning	98.0	0.90	0.03	0.84 - 0.96	6.6	27.83	< 0.001
Ventilation	98.8	0.87	0.03	0.80 - 0.93	3.2	26.85	< 0.001
Noise	98.2	0.86	0.03	0.80 - 0.93	4.8	26.74	< 0.001
Cleaning	98.8	0.77	0.03	0.71 - 0.84	1.9	23.97	< 0.001
Chemicals	98.8	0.77	0.03	0.71 - 0.84	1.9	23.97	< 0.001
Electricity	98.7	0.66	0.03	0.60 - 0.73	1.3	20.53	< 0.001
Construction	99.1	0.87	0.03	0.80 - 0.93	2.4	26.84	< 0.001
Space	96.6	0.80	0.03	0.73 - 0.86	5.9	24.63	< 0.001
***Materials***							
Facilities	96.9	0.63	0.03	0.57 - 0.70	2.9	19.59	< 0.001
Media	99.3	0.94	0.03	0.88 - 1.00	3.8	29.20	< 0.001
Furniture	99.1	0.84	0.03	0.78 - 0.90	2.0	25.99	< 0.001
Protection	98.7	0.71	0.03	0.65 - 0.77	1.5	22.00	< 0.001
***Journey/access***							
Journey	99.3	0.94	0.03	0.88 - 1.00	3.8	29.20	< 0.001
Access	99.4	0.75	0.03	0.69 - 0.81	0.8	23.17	< 0.001
**Organizational**							
***Work hours***							
Excess	94.4	0.66	0.03	0.60 - 0.72	5.8	20.41	< 0.001
Distribution	95.3	0.73	0.03	0.66 - 0.79	6.0	22.46	< 0.001
Deadlines	98.5	0.90	0.03	0.83 - 0.96	5.2	27.72	< 0.001
Workday	97.8	0.86	0.03	0.79 - 0.92	5.3	26.49	< 0.001
***Structural***							
Form	93.5	0.66	0.03	0.60 - 0.72	6.9	20.40	< 0.001
Management	94.4	0.78	0.03	0.72 - 0.85	9.6	24.28	< 0.001
Decisions	97.9	0.84	0.03	0.78 - 0.91	4.9	26.13	< 0.001
Insecurity	95.9	0.87	0.03	0.81 - 0.93	11.9	26.98	< 0.001
Incentives	97.5	0.84	0.03	0.78 - 0.91	5.6	26.12	< 0.001
Control	96.2	0.81	0.03	0.74 - 0.87	6.9	24.94	< 0.001
***Functional***							
Administrative	96.5	0.85	0.03	0.79 - 0.92	8.4	26.39	< 0.001
Role conflicts	93.8	0.64	0.03	0.58 - 0.70	6.1	19.79	< 0.001
Role ambiguities	95.0	0.78	0.03	0.72 - 0.85	8.4	24.30	< 0.001
Changes	97.5	0.66	0.03	0.60 - 0.72	2.5	20.39	< 0.001
Education	99.1	0.83	0.03	0.77 - 0.89	1.9	25.72	< 0.001
Routine	98.1	0.90	0.03	0.83 - 0.96	6.4	27.73	< 0.001
***Interpersonal***							
Competitiveness	96.9	0.78	0.03	0.71 - 0.84	4.9	24.05	< 0.001
Work environment	92.8	0.80	0.03	0.74 - 0.86	13.9	24.76	< 0.001
Harassment	97.2	0.76	0.03	0.70 - 0.82	4.1	23.53	< 0.001
Irresponsibility	97.1	0.86	0.03	0.80 - 0.93	7.3	26.69	< 0.001
**Individual**							
***Workload***							
Volume	91.9	0.68	0.03	0.62 - 0.75	9.5	21.20	< 0.001
Psychological distress	93.8	0.85	0.03	0.79 - 0.91	16.5	26.29	< 0.001
Somatic symptoms	97.5	0.89	0.03	0.83 - 0.96	8.4	27.64	< 0.001
***Powerlessness***							
Uncertainty	92.9	0.63	0.03	0.57 - 0.69	7.0	19.55	< 0.001
Contingencies	96.6	0.60	0.03	0.53 - 0.66	2.9	18.45	< 0.001
Balancing	96.5	0.83	0.03	0.77 - 0.89	7.2	25.66	< 0.001
***Rewards***							
Recognition	94.7	0.84	0.03	0.77 - 0.90	12.3	25.90	< 0.001
Remuneration	98.1	0.87	0.03	0.81 - 0.93	5.1	26.99	< 0.001
***Negligence***							
Discouragement	93.2	0.72	0.03	0.65 - 0.78	8.9	22.19	< 0.001

% = percentage of agreement between the 3 referees; k = generalized kappa (3 referees and 2 classification categories); SE = standard error; z (p) = contrast test relative to random classifier; 95% CI = confidence interval at 95%; prop = percentage of positive allocations to total allocations as a measure of prevalence. Global agreement between the 3 referees = 96.8%. Global Kappa =0.80 (95% CI = 0.79-0.81). Kappa homogeneity test: χ^2^ = 421.43 (df = 43); p < 0.001.

### Physical environment

Causes of discomfort due to physical environment conditions included setting aspects, material conditions, and journey to work and access problems.

#### Setting aspects

These included **lighting** problems, such as “*excessive artificial light and glare*” (ASS, female, 46 years) or “*terrible lighting conditions*” (TRS, male, 45 years); **air conditioning** problems, such as an “*overly dry environment, with excessive cooling and heating*” (ASS, female, 46 years); **ventilation** issues with “*unpleasant smells*” (TRS, female, 38 years) or a “*complete absence of ventilation*” (ASS, female, 46 years); **chemicals**, such as working with “*solvents*” (INT, female, 28 years), “*very aggressive chemical products*” (ASS, female, 29 years), or “*chemical compounds that pose a certain degree of danger to health*” (TRS, female, 26 years); **electrical** issues, such as being “surrounded by electric*al* devices” (INT, female, 27 years) or “*static electricity*” (ASS, female, 51 years); **noise** issues, such as “*a noisy office*” (TRS, female, 38 years) or general “*ceaseless noise*” (ASS, female, 42 years); **environmental hygiene** issues, such as “*insufficient cleaning*” (ASS, female, 46 years) or “*a lot of dust*” (TRS, female, 40 years); **construction work**, such as being “*surrounded by construction*” (TRS, male, 31 years) or “*refurbishing the building*” (ASS, male, 31 years); and **space** limitations, such as “*very small and shared, making it impossible to store important documents or books*” (INT, female, 37 years) with “*difficulties with privacy*” (TRS, female, 46 years).

#### Material conditions

These included discomfort associated with the **facilities**, which included the “*appalling state of some of the facilities*” (TRS, female, 31 years), “*working in a prefabricated building, with leaks*” (ASS, female, 31 years) with “*poor facilities*” (TRS, female, 51 years) or facilities so “*aged and deteriorated that it prevents one from working in comfort*” (ASS, female, 46 years); technical **media**, which included “*not having the adequate means to perform the job, due to budget constraints*” (ASS, male, 47 years), owing to the “*lack of resources, and due to the state and age of the existing resources*” (ASS, male, 52 years); **furniture**, which was caused by “*lack of furniture*” (TRS, male, 32 years), or because “*the furniture is not adequate for maintaining good posture: the tables, chairs…*” (TRS, female, 28 years); and personal **protective** equipment, which included a “*lack of safety in the labs: there are no lab coats provided, insufficient numbers of gloves, masks are missing…*” (TRS, female, 28 years), because “*collective protective equipment should be improved to minimize the risks*” (ASS, female, 29 years).

#### Journey to work and access problems

This category referred to journeys, as “*having to get to three different places within a period of five hours*” (TRS, female, 53 years), and **access** problems, such as the “*difficulty of accessing the workplace for research purposes outside of class hours*” (TRS, female, 36 years).

### Organizational characteristics

The organizational causes of discomfort included aspects related to working hours, organizational structure, job functions and interpersonal relations.

#### Working hours

This category included **excessive** working hours with “*long workdays*” (TRS, male, 40 years); irregular **distribution** due to the “*sudden accumulation of tasks, with very intense peaks in work*” (ASS, female, 45 years); **deadlines** due to the “*urgent nature of certain jobs*” (ASS, female, 38 years); and the type of **workday** because “*owing to the shifts we don’t have a stable schedule*” (ASS, female, 48 years).

#### Structural

These factors included the **form** of the organizational structure, with “*too many overly entrenched hierarchies unable to provide opinions and propose improvements*” (ASS, female, 29 years); due to the “*feudal structure of the departments: some people are ‘protected’ and enjoy more comfortable teaching, with more time to do research and promote themselves*” (TRS, female, 35 years), because “*the university structure is not well adapted to teamwork and collaboration*” (ASS, male, 42 years).

Structural factors included problems with **management**, given the “*lack of interest shown by superiors with respect to the department, which produces a sense of anarchy and disorganization*” (ASS, male, 34 years), the “*bosses’ lack of clear directives, disinterest and lack of motivation*” (ASS, male, 38 years), the “*lack of sufficient involvement by bosses in solving problems*” (ASS, female, 44 years) and the “*lack of understanding shown by superiors*” (ASS, male, 44 years).

Also included was **decision-making**, which was referenced by the “*lack of job autonomy*” (ASS, male, 48 years), or by finding “*too little flexibility with which to be able to carry out teaching and research work*” (TRS, female, 56 years).

Another factor was the **insecurity** because of the precarious nature of the contracts, due to the “*established working schedule, although I am a research intern and not entitled to social security*” (INT, female, 28 years), the “*job insecurity of the non-permanent staff regarding the possibilities of steady employment*” (TRS, female, 36 years) or because “*I am currently unemployed, which is to say that I have a full-time job that provides me with no income*” (INT, female, 28 years).

The scarcity of **incentives** referred to the “*absence of work promotions*” (ASS, female, 57 years), due to the “*very tough requirements in order to be promoted*” (TRS, female, 41 years), or in general because of “*slow promotions*” (TRS, male, 39 years).

The **control** mechanisms included the “*vulnerability to people with a more senior working status, who may even appropriate your work and deny support in key moments, even to save your job*” (TRS, female, 36 years), “*bosses who spend project money on personal expenses, not leaving money for necessary spending*” (INT, male, 24 years), the “*choosing of acquaintances over the best-trained people*” (ASS, male, 35 years), “*exceptions in applying rules*” (ASS, female, 47 years) and “*committees which establish rules at will*” (TRS, male, 38 years), along with making “*decisions that clearly bend the rules*” (ASS, male, 54 years).

#### Functional

These included **administrative** problems due to “*difficulties in a system with frequent opacity in the procedures*” (TRS, female, 36 years), or the “*excessive workload of administrative tasks not related to the job*” (TRS, female, 48 years), along with “c*old and distant administration of the workers and their services*” (ASS, female, 49 years).

Also included were **role conflicts**, which were due to the “*excessive time-wasting in meetings and committees which end up affecting the actual job commitment*” (TRS, male, 44 years), “*too large a teaching workload and little time for research*” (TRS, male, 50 years), “*excessive assistance workload which does not leave much time for other important tasks*” (ASS, female, 47 years) or “*dual dependence on institutions with divergent objectives*” (TRS, male, 66 years).

**Role ambiguities** were attributable to the “*lack of specified functions which correspond to the job*” (ASS, male, 34 years), “*having to take on responsibilities which are not within my scope*” (TRS, female, 31 years), the “*absolute lack of defined objectives*” (TRS, male, 25 years), the “*lack of clear guidelines*” (ASS, male, 38 years) or “*undefined tasks determined by authorities who do not know the work environment*” (ASS, female, 46 years).

The **changes** referred to the “*transformations in the university system, with new study plans which are worse than the previous plans*” (TRS, female, 32 years), “*uncertainty about the future degrees, with a small, changing and under-qualified teaching staff*” (TRS, female, 51), the “*diversity of subjects which can vary from year to year*” (TRS, female, 45 years) or the “*constant changes in functions*” (ASS, female, 41 years).

Aspects linked to **education** were identified based on the perception of a “*low educational level*” (ASS, male, 32 years) with “*needs for continued self-learning*” (ASS, male, 41 years), because “each person has to learn *when and where* he can” (ASS, female, 37) due to the “lack of experience and specialization” (TRS, male, 24 years).

**Routine** included “*monotonous tasks*” (ASS, male, 38 years), because the work is perceived as somewhat “*mechanical*” (INT, male, 27), “*routine and unrewarding*” (ASS, female, 38 years) and “*boring and monotonous*” (INT, male, 20 years) due to its “*repetitiveness*” (TRS, male, 46 years) and the “*lack of rotation in assigned tasks*” (ASS, female, 37 years) in a “*daily routine which accentuates the lack of stimulus*” (ASS, male, 51 years).

#### Interpersonal

These themes included **competitiveness**, such as “*extreme competition, even among permanent staff*” (TRS, female, 46 years), “*having colleagues who want to climb the ranks at any cost*” (TRS, female, 46 years), along with a large dose of “*individualism*” (TRS, male, 50 years).

It also included the **work environment**, due to the “*lack of support and communication between peers*” (ASS, male, 34 years), the “*rejection and contempt by some colleagues*” (ASS, male, 51 years), who “*at times are unbearable*” (ASS, male, 35 years), and the generation of “*interpersonal tensions*” (ASS, male, 28 years), “*a bad working environment, distrust*” (ASS, male, 52 years), “*bad relations between colleagues*” (ASS, male, 43 years) and “*isolation and unsatisfactory interactions*” (TRS, male, 40 years), in a “*pessimistic and judgmental human environment*” (TRS, male, 51 years), that is full of “*conflicts*” (TRS, male, 44 years), all of which causes “*easy things to become more complicated due to difficulties of dealing with people*” (ASS, male, 41 years).

**Workplace harassment** was described as “*arrogant treatment by some of the professors*” (ASS, female, 49 years), with “*disrespectful behavior*” (INT, female, 27 years), “*being looked down on by the person responsible*” (TRS, female, 31 years), being “*at times denigrated and insulted for being a woman*” (TRS, female, 51 years), and “*xenophobia from some people because I am a foreigner*” (INT, male, 29 years), along with the “*constant harassment by the dean’s office to those who are outside their circle or did not vote for this dean*” (TRS, female, 59 years).

**Irresponsibility** included the “*negative attitude of some colleagues towards their work responsibilities*” (TRS, male, 56 years), “*unmotivated staff who are not devoted to their work*” (ASS, male, 37 years) and “*colleagues with tenure who are reluctant to work and do not perform all of their functions*” (ASS, male, 29 years), because they “*try to do as little of their work as possible*” (TRS, female, 43 years), being that “*all of the work which is not done by a colleague is passed on to another person*” (ASS, female, 40 years).

### Individual conditions

The causes of discomfort due to individual conditions included aspects related to the excessive workload, feelings of powerlessness and personal negligence.

#### Workload

This included **excessive volume** due to “*excessive demands*” (ASS, male, 28 years), “*significant volume of work*” (ASS, female, 38 years), “*excess information*” (TRS, female, 37 years) and “*a lot of effort*” (TRS, female, 28 years) in a “*professional career which requires a great deal of effort*” (TRS, female, 28 years), in which an “*excessive number of students*” are served (TRS, female, 47 years) and one must “*combine talking on the phone with other activities*” (ASS, female, 33 years).

**Psychological distress** referred to “*frequent stress*” (TRS, male, 56 years), feelings of “*pressure*” (TRS, female, 46 years), “*irritability*” (TRS, male, 44 years), “*being nervous*” (ASS, female, 41 years), “*anxiety*” (TRS, female, 37 years), “*tension*” (INT, male, 29 years), “*loss of attention and concentration*” (ASS, female, 45 years), “*worse performance and rage*” (INT, female, 28 years), “*exhaustion*” (TRS, female, 43 years), “*disappointment*” (TRS, female, 46 years), “*blockage and emotional exhaustion*” (ASS, female, 48 years), which are “*depressing*” (ASS, male, 33 years) and cause “*burnout*” (ASS, female, 58 years).

The **somatic symptoms** mentioned were “*insomnia*” (TRS, female, 27), “*gastric problems*” (ASS, male, 40 years), “*headache*” (ASS, female, 43 years), “*migraines*” (TRS, female, 48 years), “*skin alterations*” (ASS, female, 48 years), “*backaches*” (ASS, female, 50 years), “*neck contractures*” (ASS, female, 33 years), “leg pain and *eye fatigue*” (ASS, female, 37 years), dysfunction in “*hearing levels*” (ASS, female, 51 years) and in general, “*an important physical toll*” (INT, female, 25 years) with “*health implications*” (ASS, female, 51 years).

#### Powerlessness

**Uncertainty** referred to the “*impossibility of looking to the future with guarantees*” (ASS, male, 31 years), due to it “*being difficult to make plans given the job instability*” (TRS, male, 45 years) or “*the many years needed to achieve a minimum level of stability (if you achieve this!), with excessive sacrifices and losses along the way*” (TRS, female, 33 years).

The **contingencies** alluded to the “*impression that whatever you do it doesn’t matter*” (ASS, female, 49 years), moreover, “*if you fail, they will talk to you, but if you do things very well, nothing happens*” (ASS, female, 41 years), and “*the more you do, the more they boss you around*” (ASS, female, 40 years), because “*it doesn’t really matter if the job is done well, badly, or not at all*” (ASS, male, 50 years).

Included in **balancing** were “*taking work issues home*” (ASS, female, 47 years), which makes you “*lack personal time*” (TRS, female, 61 years), as well as a “*difficult balance between work and family activities*” (TRS, female, 61 years).

#### Rewards

This included the absence of **recognition** due to a “*lack of appreciation*” (TRS, female, 36 years), a “*lack of recognition of the work done*” (TRS, male, 55 years) and a “*lack of social recognition*” (TRS, female, 44 years) because “*your merits and work effectiveness are not valued*” (ASS, male, 38 years). Also included was the subject of **remuneration**, such as “*a low salary that is not consistent with the professional qualifications*” (TRS, female, 33 years).

#### Negligence

This referred to individual **discouragement** due to a “*lack of enthusiasm*” (ASS, female, 48 years), and “*lower interest and certain adjustments*” (ASS, female, 50 years), of which one “*consequence is growing discouragement*” (ASS, male 51 years) and another is “*less effort*” (TRS, male, 39 years). This “*affects performance and efficacy at work*” (ASS, female, 51 years) because “*it’s not worth making the effort or doing things well*” (ASS, male, 50 years).

### Concordance between the referees

Table 1 shows the concordance of judgments according to the emerging classification system for the lower-level categories. The general agreement among the three referees was 96.8%. The obtained kappa coefficients were good in all of the codes, with values between 0.60 (contingencies) and 0.94 (technical media). Significant differences were observed between the kappa coefficients (χ^2^ = 421.43; df = 43; p < 0.001). The global kappa value was 0.80 (95% CI = 0.79-0.81). The most common complaints were related to psychological distress (16.5%), work environment (13.9%), the absence of recognition (12.3%) and insecurity (11.9%). Generally, the least common causes of discomfort were those related to the physical environment.

### Associations with the burnout types

Tables 2, 3 and 4 show the results of the bivariate analysis along with the causes of discomfort that resulted from CA and their associations with the burnout subtypes. The frenetic was associated with the presence of complaints in general (OR = 2.08; p = 0.030), facilities (OR = 3.75; p = 0.038), form (OR = 3.05; p = 0.030) and discouragement (OR = 0.14; p = 0.045). The under-challenged presented with complaints related to form (OR = 3.01; p = 0.032), incentives (OR = 5.54; p = 0.003), ambiguities (OR = 3.39; p = 0.005), routine (OR = 6.33; p < 0.001) and discouragement (OR = 2.77; p = 0.025). The worn-out was associated with complaints in general (OR = 2.97; p = 0.002), form (OR = 3.20; p = 0.024), management (OR = 6.09; p < 0.001), incentives (OR = 3.30; p = 0.030), control (OR = 13.38; p < 0.001), work environment (OR = 2.46; p = 0.010), irresponsibility (OR = 3.99; p = 0.005), contingencies (OR = 9.74; p = 0.043) and recognition (OR = 2.76; p = 0.006).

**Table 2:** Answers to the open question and causes of workplace discomfort related to the physical environment associated with the burnout types

	**Frenetic type**		**Under-challenged type**		**Worn-out type**	
**Subjects**	**Raw OR**	***p***	**Raw OR**	***p***	**Raw OR**	***p***
	**(95% CI)**		**(95% CI)**		**(95% CI)**	
***Answer***	2.08 (1.07-4.04)	**0.030**	1.54 (0.83-2.85)	0.171	2.97 (1.50-5.86)	**0.002**
**Environmental**						
***Lighting***	<0.01 (<0.01- < 0.01)	0.999	2.34 (0.62-8.87)	0.213	1.92 (0.51-7.28)	0.338
***Ventilation***	0.64 (0.16-2.99)	0.566	2.45 (0.73-8.20)	0.147	2.93 (0.88-9.80)	0.081
***Electricity***	<0.01 (<0.01- < 0.01)	0.999	2.89 (0.40-20.79)	0.292	2.38 (0.33-17.12)	0.388
***Cleaning***	2.20 (0.49-10.02)	0.306	2.17 (0.48-9.88)	0.315	3.22 (0.71-14.62)	0.130
***Space***	1.03 (0.36-2.95)	0.950	1.02 (0.36-2.91)	0.971	2.22 (0.88-5.61)	0.093
***Air conditioning***	1.48 (0.58-3.77)	0.413	1.15 (0.43-3.05)	0.779	1.19 (0.47-3.03)	0.714
***Chemicals***	0.48 (0.06-4.01)	0.495	2.17 (0.48-9.88)	0.315	3.22 (0.71-14.62)	0.130
***Noise***	1.64 (0.54-5.01)	0.387	1.15 (0.35-3.74)	0.820	1.81 (0.61-5.34)	0.282
***Construction***	1.76 (0.41-7.89)	0.446	1.73 (0.41-7.39)	0.457	0.78 (0.16-3.94)	0.766
**Materials**						
***Facilities***	3.75 (1.07-13.17)	**0.038**	0.81 (0.17-3.97)	0.796	1.92 (0.51-7.28)	0.338
***Media***	1.30 (0.39-4.31)	0.671	1.28 (0.39-4.25)	0.688	0.70 (1.19-2.59)	0.593
***Furniture***	0.57 (0.07-4.97)	0.614	0.57 (0.07-4.91)	0.606	0.47 (0.05-4.04)	0.490
***Protection***	<0.01 (<0.01- < 0.01)	0.999	<0.01 (<0.01- < 0.01)	0.999	<0.01 (<0.01- < 0.01)	0.999
**Journey/access**						
***Journey***	0.72 (0.15-3.43)	0.677	0.71 (0.15-3.39)	0.665	1.59 (0.44-5.75)	0.477
***Access***	<0.01 (<0.01- < 0.01)	0.999	<0.01 (<0.01- < 0.01)	0.999	4.78 (0.43-53.27)	0.203

Raw OR: odds ratio resulting from bivariate analysis through a logistic regression model. CI: confidence interval. ‘Answer’ refers to the fact of a general answer to the open question with any complaint, as opposed to not giving any answer to this question. Significant values (p < 0.05) are in bold.

**Table 3:** Causes of workplace discomfort resulting from the organizational environment associated with the burnout types

	**Frenetic**		**Under-challenged**		**Worn-out**	
**Subjects**	**Raw OR**	***p***	**Raw OR**	***p***	**Raw OR**	***p***
	**(95% CI)**		**(95% CI)**		**(95% CI)**	
**Work hours**						
***Excess***	2.57 (0.84-7.84)	0.097	0.51 (0.11-2.34)	0.385	0.42 (0.09-1.92)	0.263
***Distribution***	0.71 (0.20-2.58)	0.608	1.04 (0.32-3.34)	0.949	0.35 (0.08-1.58)	0.174
***Deadlines***	0.66 (0.18-2.35)	0.519	0.95 (0.30-3.01)	0.929	0.53 (0.15-1.90)	0.332
***Workday***	0.19 (0.02-1.42)	0.104	1.76 (0.62-4.96)	0.288	0.53 (0.15-1.90)	0.332
**Structural**						
***Form***	3.05 (1.12-8.36)	**0.030**	3.01 (1.10-8.24)	**0.032**	3.20 (1.16-8.82)	**0.024**
***Management***	1.31 (0.55-3.11)	0.541	1.56 (0.67-3.62)	0.300	6.09 (2.57-14.45)	**<0.001**
***Decisions***	0.23 (0.03-1.82)	0.165	2.54 (0.83-7.73)	0.102	0.70 (0.19-2.59)	0.593
***Insecurity***	1.38 (0.67-2.85)	0.384	0.74 (0.33-1.67)	0.470	1.10 (0.53-2.26)	0.798
***Incentives***	1.64 (0.54-5.01)	0.387	5.54 (1.81-16.93)	**0.003**	3.30 (1.12-9.74)	**0.030**
***Control***	2.43 (0.93-6.33)	0.070	1.88 (0.71-4.98)	0.206	13.38 (3.80-47.19)	**<0.001**
**Functional**						
***Administrative***	0.71 (0.26-1.94)	0.502	0.70 (0.26-1.91)	0.485	0.91 (0.37-2.25)	0.841
***Conflicts***	1.99 (0.69-5.73)	0.204	1.04 (0.32-3.34)	0.949	0.35 (0.08-1.58)	0.174
***Ambiguities***	0.59 (0.20-1.79)	0.352	3.39 (1.45-7.96)	**0.005**	2.28 (0.98-5.33)	0.057
***Transformations***	0.57 (0.07-4.97)	0.614	0.57 (0.07-4.91)	0.606	2.40 (0.48-12.04)	0.289
***Education***	0.72 (0.08-6.52)	0.770	0.71 (0.08-6.43)	0.761	0.59 (0.07-5.30)	0.635
***Routine***	0.82 (0.26-2.55)	0.730	6.33 (2.31-17.36)	**<0.001**	0.28 (0.06-1.25)	0.096
**Interpersonal**						
***Competitiveness***	2.34 (0.85-6.46)	0.100	0.40 (0.09-1.77)	0.225	0.33 (0.07-1.46)	0.142
***Work environment***	1.44 (0.70-2.99)	0.327	1.63 (0.79-3.33)	0.184	2.46 (1.24-4.88)	**0.010**
***Harassment***	1.46 (0.36-5.95)	0.598	0.81 (0.17-3.97)	0.796	3.04 (0.80-11.51)	0.103
***Irresponsibility***	1.12 (0.39-3.22)	0.836	2.39 (0.92-6.24)	0.075	3.99 (1.51-10.56)	**0.005**

Raw OR: odds ratio resulting from bivariate analysis through a logistic regression model. CI: confidence interval. Significant values (p < 0.05) are in bold.

**Table 4:** Causes of workplace discomfort based on individual conditions associated with the burnout types

	**Frenetic**		**Under-challenged**		**Worn-out**	
**Subjects**	**Raw OR**	***p***	**Raw OR**	***p***	**Raw OR**	***p***
	**(95% CI)**		**(95% CI)**		**(95% CI)**	
**Overload**						
***Volume***	1.29 (0.51-3.22)	0.593	1.27 (0.51-3.17)	0.614	1.03 (0.41-2.58)	0.943
***Distress***	1.20 (0.60-2.39)	0.603	0.69 (0.32-1.48)	0.335	1.68 (0.88-3.18)	0.114
***Symptoms***	1.21 (0.48-3.00)	0.689	0.39 (0.11-1.34)	0.134	1.21 (0.48-3.00)	0.689
**Powerlessness**						
***Uncertainty***	2.10 (0.78-5.67)	0.143	0.60 (0.17-2.13)	0.429	0.72 (0.23-2.24)	0.567
***Contingencies***	0.72 (0.08-6.52)	0.770	1.92 (0.32-11.66)	0.478	9.74 (1.08-88.05)	**0.043**
***Balancing***	0.47 (0.13-1.62)	0.229	0.89 (0.32-2.48)	0.818	0.54 (0.18-1.64)	0.276
**Rewards**						
***Recognition***	1.50 (0.70-3.21)	0.297	0.75 (0.32-1.79)	0.517	2.76 (1.34-5.68)	**0.006**
***Remuneration***	1.33 (0.45-3.93)	0.605	0.65 (0.18-2.32)	0.505	0.78 (0.25-2.47)	0.671
**Negligence**						
***Discouragement***	0.14 (0.02-1.02)	**0.045**	2.77 (1.14-6.73)	**0.025**	1.19 (0.47-3.03)	0.714

Raw OR: odds ratio resulting from bivariate analysis through a logistic regression model. CI: confidence interval. Significant values (p < 0.05) are in bold.

The multivariate analysis for the frenetic showed correlations with complaints (OR = 2.43; CI = 1.21-4.90; p = 0.013), form (OR = 4.32; CI = 1.43-13.06; p = 0.010) discouragement (OR = 0.12; CI = 0.02-0.92; p = 0.041). The model fit was acceptable (χ^2^ = 5.85; df = 8; p = 0.664) and 75.8% of the cases were classified correctly (ROC = 0.72; 95% CI = 0.66-0.77; p < 0.001). The under-challenged correlated with form (OR = 3.05; CI = 1.06-8.84; p = 0.039), incentives (OR = 4.14; CI = 1.25-13.67; p = 0.020), ambiguities (OR = 3.61; CI = 1.50-8.70; p = 0.004) routine (OR = 5.33; CI = 1.84-15.40; p = 0.002). The fit was acceptable (χ^2^ = 11.19; df = 8; p = 0.191) and 75.8% of the cases were classified correctly (ROC = 0.68; 95% CI = 0.62-0.75; p < 0.001). The worn-out correlated with management (OR = 3.75; CI = 1.44-9.75; p = 0.007), control (OR = 6.13; CI = 1.57-23.91; p = 0.009) recognition (OR = 2.47; CI = 1.14-5.34; p = 0.021). The fit was acceptable (χ^2^ = 5.14; df = 8; p = 0.742) and 76.8% of the cases were classified correctly (ROC = 0.74; 95% CI = 0.68-0.79; p < 0.001).

## Discussion

This study is the first to analyze the potential associations between the causes of workplace discomfort, which were openly and freely expressed by workers, and distinct subtypes of burnout in a large work organization such as a university. Therefore, a mixed study approach that integrated both qualitative and quantitative research methods was required. This approach allowed us to collect the workers’ complaints in their own words, and to estimate the risks posed by the presence of certain issues in cases of high scores in the different burnout profiles. In general, this study resulted in an extensive list of negative experiences that can affect employees in the workplace, and has improved our understanding of the particular idiosyncrasies that underlie the different burnout subtypes.

With regard to study limitations, we must mention that the participant answers were self-reported and therefore might be influenced by social desirability and possible subsequent attenuation. It is also possible that some specific burnout types are biased, from a cognitive standpoint, when it comes to identifying specific causes of discomfort in their setting. Nevertheless, we are dealing with subjective perceptions, and the actual symptoms of burnout may lead to a view that is different from reality. In this regard, the idea of reverse causality cannot be ruled out completely. Moreover, although the use of an open question in a broad sample permitted the sampling of a large content area, it limited the conceptual depth of the responses and the possible interpretations due to the lack of a deeper approach. In order to achieve a deeper understanding of the results, it would be advisable to conduct future studies of a purely qualitative nature, using the topics arising in this study as a list of contents for specific investigation. Finally, the response rate obtained was low, although it was within expectations when the data collection process was taken into account [38,39]. It should also be added that the overload affecting the frenetic subtype and the neglect affecting the worn-out subtype could have led these subtypes to be more hesitant to participate [33], which would reduce the representativeness of the sample. With regard to study strengths, we refer to the broad multi-occupational sample of employees in a very diverse range of jobs who were at risk of developing burnout due to the nature of workplace personal contacts. This diversity allowed us to expand the possible generalizations of our results. The concordances between the referees were generally very high, which lent credibility to the coding process and the category system itself in the representation of the open responses. Furthermore, the data quality was controlled by eliminating possible errors in the transcription process using software specifically designed for this purpose.

We observed the following three major discomfort frameworks within an academic work organization: the physical environment, the organizational characteristics and the individual conditions. The physical environment included setting aspects, material issues and work approximations. The organizational characteristics included references to the working schedule, the organizational structure, job functions and interpersonal relations. Meanwhile, individual conditions included those caused by excessive workload, the feeling of powerlessness, the absence of rewards and negligence at a personal level.

The relatively low prevalence of the lower-level categories could have possibly facilitated the high level of agreement achieved among the three referees. We observed a higher level of agreement in the identification of discomfort caused by lack of material resources and journeys to work. In contrast, the agreement was lower for subjects such as uncertainty about the future and the system of contingencies. This observation suggests that issues related to the physical environment were less conflict-prone and were perhaps expressed in a clearer manner. Nevertheless, we observed that complaints related to the physical environment were generally less frequent in comparison with aspects such as psychological distress and the absence of recognition, at the individual level, or insecurity and the work environment, at the organizational level. Discomfort caused by organizational characteristics exhibited the highest degree of correlation with the burnout profiles. Specifically, this discomfort originated as a consequence of structure, because of the hierarchical form, lack of incentives, management, and control mechanisms, and was also caused by functions, due to routine tasks and role ambiguities. We also found important correlations between the burnout profiles and individual conditions, due to the lack of rewards and negligence. In contrast, the physical environment barely played a role in this regard.

The frenetic burnout profile was highlighted by giving answers to the open question in general, and was characterized to indicate the form of the organizational structure, as an important cause of discomfort, along with the absence of discouragement at a personal level. In other words, although the frenetic profile is highly involved and committed, such people openly report experiences of discomfort at the workplace. It is noteworthy that the type of difficulties experienced by this profile do not involve deteriorating health, which is generally indicated for burnout [49,50] and which would be expected for this profile, due to the work overload [32-35,51]. However, this subtype seems to be specifically upset by the form of the organization. It is precisely the organizational structure —the hierarchy it constructs and the possible injustices derived from the situation— that is crucial to the generation of dissatisfaction and, ultimately, to the development of burnout [52-54]. The mechanism by which this affects the frenetic subtype could be the failure to meet expectations with regard to personal aspirations and ambition, which are specifically present in this profile [27]. Nevertheless, this is a profile that seems to be resistant to the loss of personal interest, perhaps due to the intrinsic origin of work motivation [55].

The under-challenged subtype reported discomfort at work resulting from a lack of incentives for professional promotion at the level of the organizational structure, as well as functional role ambiguities and routines. These results indicate workplace tasks as the foremost cause of dissatisfaction in this burnout profile, which is consistent with the findings of previous studies [3,33]. Role ambiguity due to insufficient task definition has been described as an important factor associated with the development of burnout; this factor can predict burnout development with three years of anticipation [56-58]. Moreover, routine tasks that cause tediousness and boredom can lead to a lack of stimulus and growth in the workplace [59], which could be avoided by supervised work plans that are intended to enhance creativity [60]. It is believed that the absence of development opportunities at work and growth within the company is associated with burnout due to the absence of personal fulfillment [61]. Furthermore, burnout seems to be dynamic with regard to internal workplace changes, such as promotions or lateral job transitions [62]. In other words, burnout development could possibly be reduced by such changes. These causes of dissatisfaction, which are associated with the under-challenged profile, permit an understanding of the absence of enthusiasm and indifference that result from a lack of job incentives [27].

The worn-out subtype reported discomfort caused by the upper levels of the organizational structure and associated control mechanisms, along with discomfort resulting from the individual experience of a lack of rewards in the form of recognition. It has been reported that adequate supervisorial support can moderate the effects of the job demands and that the absence of such support can predict emotional exhaustion [63,64]. Furthermore, experiences of injustice or a lack of equity in applied norms have been associated with burnout in general [65,66]. Both of these features indicate that the worn-out subtype chooses to reduce his or her level of involvement up to the abandonment of responsibilities and that this choice may be due to the lack of supervisorial support in completing work tasks and the inconsistent application of norms and control mechanisms. Therefore, it is not surprising that this profile is characterized by negligence [27] or that it expresses complaints regarding the lack of recognition for expected work because as we have mentioned, these employees are not properly completing their work. The experience of the absence of recognition is a common symptom of burnout and is specifically associated with the perception of inefficacy [67,68], which agrees with our arguments.

All of these findings reinforce the idea that the development of burnout results not only from exhaustion due to an excessive workload but also from conflict processes in other significant areas of professional life [69]. The specific resolution of these conflicts in the interactive context of the person and his or her work environment might favor the development of other important symptomatic aspects of the syndrome, which are in turn based on the different developmental paths of burnout specific to the distinct subtypes. However, the pattern of the associations between the factors considered in the study requires further research to deepen our understanding of its true nature.

## Conclusions

Using a QQA or mixed methodological approach, we identified the principal causes of discomfort suffered by the employees of a large organization. The diverse motives underlying the complaints we observed can be used as a guide for the gathering of qualitative information to design assessment and intervention programs intended to alleviate discomfort in employees. The above-mentioned methodological approach also allowed us to determine the negative work experiences that affect those who suffer from one of the burnout profiles, as openly expressed in their own words. We observed that the patterns of relationships between the different complaint motives and the various profiles reinforce the burnout subtypes model; the patterns are consistent with the definitions proposed for each of the subtypes and thus provide validity to the model. The fact that the burnout subtypes are affected by different causes of discomfort suggests that the subtypes should be specifically addressed in therapy, considering the particular characteristics of each to optimize the effectiveness of interventions.

## Competing interests

The authors declare that they have no competing interests.

## Authors’ contributions

JMM and JGC designed the project. JMM collected the data. JMM, JPA, SG, JMC and AAM performed the qualitative analysis. JMM developed the statistical analysis. All authors interpreted the results, drafted the manuscript and read and approved the final manuscript.

## Pre-publication history

The pre-publication history for this paper can be accessed here:

http://www.biomedcentral.com/1471-2458/13/1240/prepub
